# Understanding the Changes in Brain Activation When Viewing Products with Differences in Attractiveness

**DOI:** 10.3390/neurolint16050069

**Published:** 2024-08-28

**Authors:** Emily L. L. Sin, Clive H. Y. Wong, Bolton K. H. Chau, Matthias Rauterberg, Kin Wai Michael Siu, Yi-Teng Shih

**Affiliations:** 1 School of Design, The Hong Kong Polytechnic University, Hong Kong; sin.emily@gmail.com (E.L.L.S.); m.siu@polyu.edu.hk (K.W.M.S.); 2 Department of Psychology, The Education University of Hong Kong, Hong Kong; hycwong@gmail.com; 3 Department of Rehabilitation Sciences, The Hong Kong Polytechnic University, Hong Kong; bolton.chau@polyu.edu.hk; 4 Mental Health Research Centre, The Hong Kong Polytechnic University, Hong Kong; 5 University Research Facility in Behavioral and Systems Neuroscience, The Hong Kong Polytechnic University, Hong Kong; 6 Department of Industrial Design, Eindhoven University of Technology, Eindhoven, The Netherlands; g.w.m.rauterberg@tue.nl

**Keywords:** brain activation, chair design, fMRI, product attractiveness, design strategies

## Abstract

Product design and attractiveness are pivotal factors that determine people’s positive reactions when viewing a product and may eventually affect their purchasing choices. Comprehending how people assess product design is crucial. Various studies have explored the link between product attractiveness and consumer behavior, but these were predominantly behavioral studies that offered limited insight into the neural processes underlying perceptions of product attractiveness. Gaining a deeper understanding of these neural mechanisms is valuable, as it enables the formulation of more objective design guidelines based on brain activity, enhancing product appeal and, ultimately, spurring consumer purchases. In our study, we sought to (1) elucidate the neural network engaged when individuals evaluate highly attractive product images, (2) delineate the neural network activated during the evaluation of less attractive product images, and (3) contrast the differences in neural networks between evaluations of highly and less attractive images. We utilized fMRI to investigate the neural activation patterns elicited by viewing product images of varying attractiveness levels. The results indicated distinct neural activations in response to the two types of attractive images. Highly attractive product images elicited activity in the anterior cingulate cortex (ACC) and the occipital pole, whereas less attractive product images stimulated the insula and the inferior frontal gyrus (IFG). The findings of this project provide some of the first insights of its kind and valuable insights for future product design, suggesting that incorporating more positive and rewarding elements could enhance product appeal. This research elucidates the neural correlates of people’s responses to product attractiveness, revealing that highly attractive designs activate reward-related brain regions, while less attractive designs engage areas associated with emotional processing. These findings offer a neuroscientific basis for further studies on developing design strategies that align with consumers’ innate preferences, potentially transforming product design and marketing practices. By leveraging this knowledge, designers can craft products that not only meet functional needs but also resonate more deeply on an esthetic level, thereby enhancing consumer engagement and purchase likelihood.

## 1. Introduction

In a competitive marketplace, businesses are shifting their focus to consumers’ emotional needs, such as esthetics, emotional preferences, and connection, rather than just focusing on basic functions [1]. Mumcu et al. [2] proposed that visual esthetics are increasingly seen as a key player in marketing and new product development among these emotional demands. As a sensory reflection of product design features, visual esthetics help differentiate a product from a competitors’ products. A product’s visual esthetics have a profound impact on increasing acceptance and stimulating purchase intentions [3]. Moreover, Simmonds and Spence [4] suggested that the visual attractiveness of the packaging can also affect consumer perception and purchasing behavior.

Making the product attractive is the goal of every designer, and it is also a big challenge. To achieve this, we need to understand how consumers feel about product design features, i.e., we need to do a deep dive into how they appreciate or interpret the design features that designers add to a product. Which design attributes of a product’s shape are important to consumer perception has important implications for how a successful product is designed, developed, produced, and marketed. Therefore, only when consumers can recognize the product design language used by designers and interpret the meaning and quality that designers give to products can they determine their value in the market.

The visual appeal of a product plays a pivotal role in shaping its brand perception and significantly impacts one’s positive reactions when viewing the product. Thus, the critical question is how to craft a product’s appearance to captivate consumers at first glance. Furthermore, it is vital to explore how design strategies can be employed to create an immediate allure for consumers. During the design phase of a product’s form, designers engage in a dynamic process where their manipulation of cognitive models gives rise to a variety of forms, imbuing the product with a range of images that linger in the consumer’s memory. The complexity of the product’s shape garners consumer focus, promotes detailed scrutiny, and fosters memorable impressions, all the while reflecting the designer’s skill and unique style [5].

Around the sensory perception of appearance, scholars use a variety of methods to measure the visual attractiveness of products. Most scholars rely on subjective reports, such as the visual attractiveness of a website’s inventory and the semantic difference scale for product appearance [6]. Subjective reports, while simple and easy to understand, do not provide users with an instant assessment of how a product looks without interrupting the flow of appreciation. As a result, few researchers have attempted to develop new methods for measuring the appearances of products. For example, Liu and MacGregor [7] integrated machine vision methods to measure product appearance using texture features extracted from product images. Mayer and Landwehr [8] designed an objective measure of a product typicality based on distances between feature points. Many scholars have focused on measuring the design characteristics of products, but very limited amounts of research focus on understanding the changes in consumer brain activity due to different levels of product attractiveness.

Recently, more and more scholars have begun to use fMRI to study brain-related design activities. fMRI research is built on an understanding of the mental processes and representations involved in design. Research in design cognition generates knowledge about the mental processes involved in the act of designing and how they interact. fMRI can, in turn, show researchers which brain regions and networks are associated with mental processing during designing [9]. However, to study design using fMRI is challenging.

The most important thing for product appearance to cause interaction is the attractiveness of the product, but the hidden factors behind the product’s attractiveness are not easy to know. Mechanisms must be used to discover the evidence that affects the attractiveness of the product. Generally, experts and scholars use the interview method. However, this method can only explore the differences in the appearances of general products. The use of fMRI provides a new, insightful approach to potentially understanding brain activity mechanisms. However, findings from neuroscience research, while insightful, are rarely directly applied to design practice. Therefore, understanding the relationship between product attractiveness and people’s brain activity is a question worthy of study. This study aims to be the first study to understand if product attractiveness will have different effects on brain activity and to provide a solid background for future study on individualized product attractiveness and consumer preferences.

## 2. Research Question

The research question for the proposed project is whether highly attractive and less attractive products stimulate people’s brain activity differently. To answer this research question, the following three research objectives are proposed:

Objective 1: Understand the brain activations when a person is assessing highly attractive product images.

Objective 2: Understand the brain activations when a person is assessing less attractive product images.

Objective 3: Compare the differences in brain activation when people are assessing highly attractive and less attractive images.

In particular, we hypothesized that there are differences in brain activation when people view highly attractive product images and less attractive product images, particularly in the areas that are related to reward processing.

## 3. Materials and Methods

In this study, we adopted the same research protocol in subject recruitment and fMRI scanning for Objectives 1–3. This study was approved by the ethics committee of the Hong Kong Polytechnic University (HSEARS20221113003).

### 3.1. Participants

A total of 46 participants with design backgrounds (24 females and 22 males) with ages ranging from 20 to 28 years old were recruited through word of mouth. Participants were only included if they did not have any history of major psychiatric or physical illness. Participants who were taking any kind of medication during the data collection period were excluded to ensure there were no confounding effects of neurological changes due to drug effects. Participants who did not meet MRI safety requirements were also excluded. Participants were briefed on the MRI scanning procedures before the MRI scan, and voluntary consent forms were then signed.

### 3.2. Stimuli Materials

**Figure 1:**
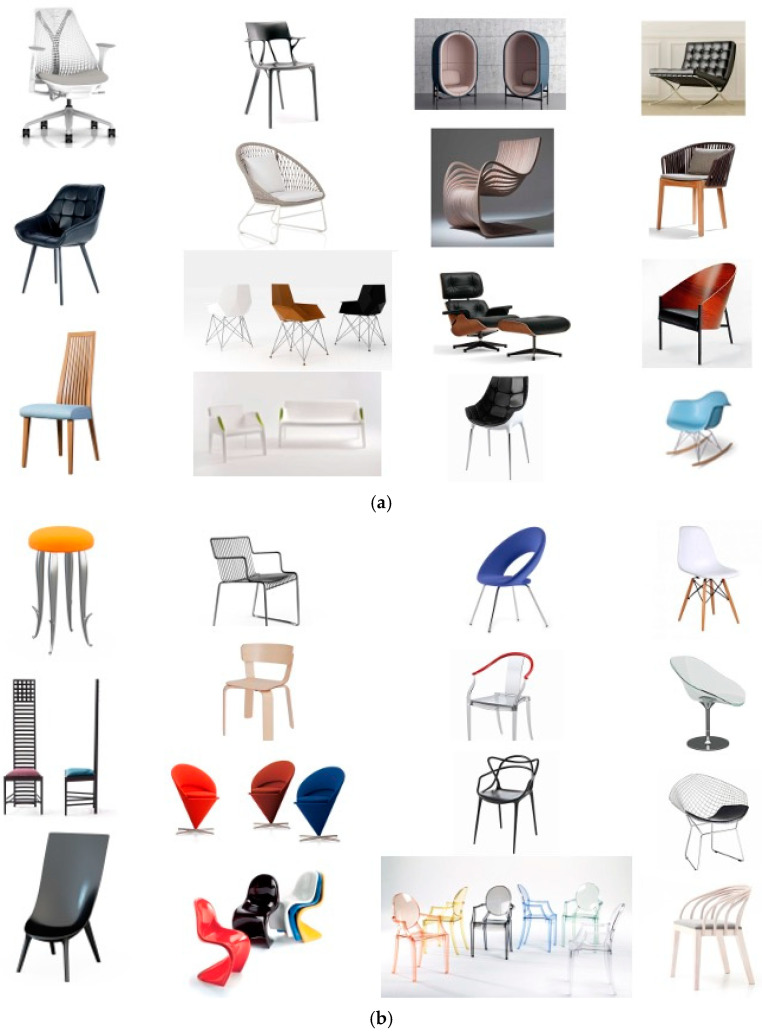
(**a**): Highly attractive pictures. (**b**): Less attractive pictures.

**Figure 2:**
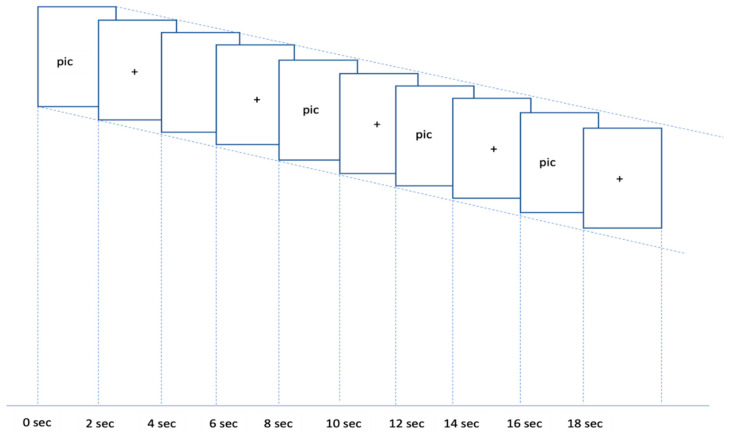
The sequence of events in a given trial.

A between-subjects experimental design was employed in the study. Two different types of attractive pictures (Figure 1a,b) were presented to the participants. In a given trial, 5 pictures were presented for a total of 20 s, including a highly/less attractive picture (2 s), a fixation (2 s), a highly/less attractive picture (2 s), a fixation (2 s), a highly/less attractive picture (2 s), a fixation (2 s), highly/less attractive picture (2 s), a fixation (2 s), a highly/less attractive picture (2 s), and a fixation (2 s), followed by a 16 s rest. A total of 12 trials (6 trials with highly attractive pictures and 6 trials with less attractive pictures) were be presented to each participant. Figure 2 illustrates an example of the sequence of events in one trial.

Highly and less attractive images were selected using the following methodology: An expert panel consisting of five individuals with a background in design was convened to choose 30 distinct chair images. Subsequently, community participants were invited to assess the appeal of these chairs through online surveys, employing a 1–5 Likert scale for their ratings. The chairs were then classified into two categories based on their attractiveness scores of high attractiveness (scores of 3 or above) and low attractiveness (scores below 3) (Table 1). Table 2a,b shows the correlation between the 3 groups of ratings. We also found that the ratings were significantly different between highly and less attractive pictures (t = 9.6179, df = 27.017, *p*-value < 0.001).

Following this categorization, Magnetic Resonance Imaging (MRI) scans were conducted to analyze the corresponding brain activity.

### 3.3. Functional MRI (fMRI) Scanning

To investigate brain activities, we employed the functional Magnetic Resonance Imaging (fMRI) scanning technique. fMRI, utilizing blood-oxygen-level dependent (BOLD) contrast, has become a staple in neuroscience research for examining functional activities and cognitive behaviors associated with specific tasks—known as task-based fMRI. fMRI generated activation maps that represented the average engagement of various brain regions during tasks or in response to certain stimuli. These maps could be compared across subjects or conditions to discern the relative intensity of different neural responses.

For our specific aims, we conducted studies in distinct phases. Following the behavioral approach for classifying picture attractiveness, task-based fMRI experiments were implemented using a block design via the online software PsychoPy (v2022.2.1). The study incorporated two different types of stimulus blocks: (i) low attractiveness and (ii) high attractiveness. In the low attractiveness condition, 15 images were displayed in 3 blocks, each in a randomized sequence. Similarly, for the high attractiveness condition, another set of 15 images was showcased in a randomized order across 3 blocks. The presentation order of the blocks was counterbalanced to mitigate any potential carryover effects. Participants were shown a fixation crosshair before each trial, serving as a rest period to reset attention before the subsequent viewing of pictures with varying levels of attractiveness. Passive viewing was employed in our study; participants did not need to do any rating of the pictures while they were in the scanner. Participants were then asked to rate the attractiveness of the pictures using the same 1–5 Likert scale after the MRI scan was finished.

### 3.4. fMRI Acquisition

Neuroimaging data were collected for each of the blocks. All images were acquired using a Siemens Magnetom 3T scanner (Siemens, Erlangen, Germany). For the functional (EPI) scan, the following acquisition parameters were used: Scan time: 7:36 min; TR: 3000 ms; TE = 30 ms; Field of View (FOV): 214 mm; Flip Angle = 85°; voxel size: 0.8 × 0.8 × 0.8 mm^3^. A T1 structural scan and a resting scan were performed before the functional scan and employed the following parameters: Scan time: 6.54 min; TR: 3000 ms; Flip Angle: 85°; TE: 30 ms; voxel size: 0.8 × 0.8 × 0.8 mm^3^.

### 3.5. fMRI Analysis

#### 3.5.1. Structural Imaging Processing

High-resolution structural T1-weighted images were acquired for each participant. Skull stripping was performed on these images using the Opti bet tool [10], which employs an optimized brain extraction technique to improve the accuracy of separating brain tissue from non-brain structures. The structural images were normalized to the MNI template with nonlinear registration using FSL/FNIRT.

#### 3.5.2. Functional Imaging Processing

We used fsl_motion_outliers to calculate framewise displacement (FD). Following the recommendations by Siegel et al. [11], volumes with an FD exceeding a threshold of 0.9 mm were flagged for censoring or scrubbing, ensuring the reduction in motion-related noise in the subsequent analysis. This conservative threshold was chosen to minimize the impact of micro-movements that are known to distort the BOLD signal, therefore improving the validity of the detected functional connectivity patterns.

#### 3.5.3. fMRI Data Preprocessing

The functional imaging data underwent several preprocessing steps using FSL software. This included temporal high-pass filtering with a 90 s cutoff to remove low-frequency drift, spatial smoothing with an 8 mm full width at half maximum (FWHM) Gaussian kernel to increase the signal-to-noise ratio, and slice-timing correction to adjust for the temporal offset between slices acquired in each volume. Functional images were co-registered to each participant’s structural image using boundary-based registration (BBR) to ensure accurate alignment.

#### 3.5.4. First-Level Modeling

First-level statistical analysis was conducted within the framework of the general linear model (GLM) as implemented in FSL/FEAT. The GLM included standard motion parameters and extended motion parameters as covariates to account for residual motion effects. Motion outliers and framewise displacement were also included as additional confound explanatory variables (EVs) to further control motion-related noise. Task-related BOLD responses were modeled using FMRIB’s Linear Optimal Basis Sets (FLOBS) with three actual EVs for each condition, capturing the evolution of the hemodynamic response [12].

#### 3.5.5. Summary Statistic Calculation

To create a summary statistic for each condition, the root mean square (RMS) value of the three parameter estimates (PEs) was computed across the condition-specific EVs for each participant [13]. These RMS values were used to represent the overall activation level in response to each condition. Subsequently, the summary statistics were transformed into the standard MNI space to facilitate group-level comparisons.

#### 3.5.6. Group-Level Analysis

A higher-level analysis was carried out using randomization, a non-parametric permutation-based inference tool. The analysis was structured as a paired *t*-test to directly compare brain responses to highly attractive images versus less attractive images within subjects. The permutation test was configured to use sign-flipping, ensuring the appropriate test for the paired design. Exchangeability blocks were defined to preserve the paired nature of the comparison across permutations. The primary contrast of interest tested the hypothesis that highly attractive images would elicit greater neural activation than less attractive images.

#### 3.5.7. Statistical Thresholding and Cluster Formation

Statistical maps generated from permutation testing were subjected to threshold-free cluster enhancement (TFCE) to identify regions of significant activation without the need for arbitrary cluster-forming thresholds [14]. The resulting TFCE-corrected *p*-values were thresholded at a *p* < 0.001 to control for family-wise error across the whole brain. Clusters were considered significant if they surpassed a t-value of >8 and encompassed a minimum of 50 contiguous voxels, ensuring the robust detection of activation while minimizing false positives.

## 4. Results

**Figure 3:**
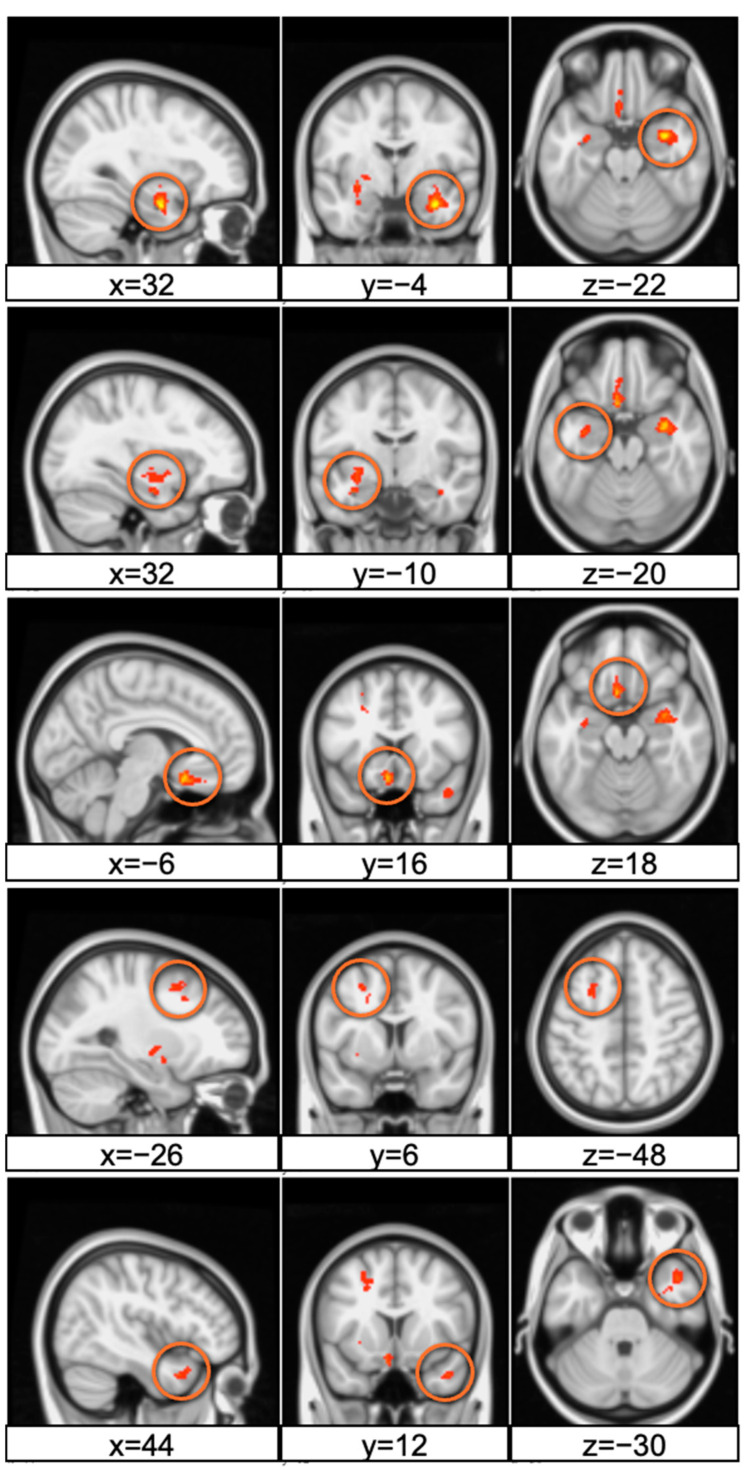
Activation map of the five clusters.

In this study, we examined the activation of different brain regions when the participants were viewing products with high attractiveness and low attractiveness. Significant brain activation clusters were identified, as summarized in Table 3. In cluster analysis, we found five significant clusters of activation that differed when participants viewed highly attractive product designs compared to less attractive ones (Figure 3). The clusters are ordered by the number of voxels they contain, with the largest cluster labeled as Cluster 5 and the smallest as Cluster 1.

Cluster 5, with 222 voxels, exhibited the strongest activation (maximum T-value = 14.7), located in the right amygdala. The smallest cluster (Cluster 1), with 65 voxels, also demonstrated significant activation with a maximum T-value of 10.3 in the right superior temporal pole. The activation clusters were anatomically localized to regions known to be involved in emotional processing and reward evaluation. Notably, the right amygdala and the left putamen are structures implicated in affective and reward-related processing, respectively. The identified clusters reached statistical significance after correcting for multiple comparisons using TCFE. The brain activation patterns elicited by high versus low attractiveness in product designs provide insights into the neural correlates of esthetic appreciation and preference. These results support the hypothesis that visual attractiveness in product design is discernible at the neural level, with specific brain regions differentially engaged during the perception of attractive designs.

## 5. Discussion

Our study found there are different activation areas when people are viewing highly and less attractive product images. To best of our knowledge, this is the first study that explores product attractiveness and brain activities. Previous studies have mainly focused on domain specific areas, like studies on valence arousal, facial attractiveness and architectural interior design [15,16]. Our study found that when people were viewing highly attractive images, the amygdala, putamen, subgenual anterior cingulate cortex, dorsolateral prefrontal cortex, and temporal pole were activated compared to when they were viewing less attractive images.

### 5.1. Amygdala

The amygdala is critical in detecting emotionally salient stimuli, whether they are positive or negative. It helps to prioritize such stimuli for processing, ensuring that emotionally relevant information is addressed [17]. When a positive stimulus is presented, the amygdala helps to spotlight rewarding and pleasant experiences, enhancing their salience and memorability. Studies have shown that emotionally salient stimuli can capture attention automatically. The amygdala plays a role in this process by enhancing the perceptual processing of emotionally significant stimuli, even when they appear outside of the center of focus of attention [18]. Also, the amygdala will interact with sensory cortices to modulate sensory processing based on emotional relevance. The amygdala will amplify the visual processing of the highly attractive images in the occipital cortex, thereby giving these images more attention during informational processing [19].

In our study, the amygdala activation in response to viewing highly attractive products can be understood through its role in processing salience and reward. Attractiveness in products is often a combination of esthetic appeal, perceived quality, and potential status enhancement, all of which can be inherently rewarding to people. As mentioned, the amygdala is involved in processing emotionally salient stimuli. Highly attractive products may evoke a positive emotional response due to their design, color, or association with pleasure, making them salient to the individual. Also, highly attractive products can trigger the brain’s reward system, of which the amygdala is also a part. The anticipatory pleasure of owning or using an attractive product activates the brain’s reward pathways, which include other neural correlates such as the nucleus accumbent and the orbitofrontal cortex. This activation can be in the same sense as other rewarding stimuli, such as food or social interaction. The activation of the amygdala in product appreciation is coherent with previous studies of the human brain’s responses to natural beauty. Previous studies have shown that the amygdala plays an important role in evaluating highly attractive faces and this finding has been extended to man-made products in our study. Viewing esthetically pleasing objects can be inherently rewarding, in accordance with their natural beauty or object recognition.

Product design is highly associated with people’s preferences. It is believed that products having higher levels of attractiveness will increase the buying tendency for those products [20,21]. The amygdala is also involved in decision-making processes, particularly those that are influenced by emotions. When evaluating products, emotional reactions can heavily influence people’s behaviors. The attractiveness of a product may evoke a strong emotional response that could guide people to perform positive behaviors that may involve the amygdala. The activation of the amygdala when viewing highly attractive products is multifaceted, involving emotional salience, reward, esthetic appreciation, and decision-making. This activation reflects the complex interplay between emotion, cognition, and behavioral intentions in response to stimuli that are perceived as attractive and desirable.

### 5.2. Putamen

The putamen is another significant cluster area that was found when the participants were viewing highly attractive product designs. The putamen is involved in different functions, including motor control, learning, and reward-related processing. When individuals view attractive products, the putamen can be implicated in different ways. The putamen is a key structure in the brain’s reward circuitry. It is involved in the processing of rewards and the reinforcement of learning that helps associate certain stimuli with positive outcomes. When a product is perceived as highly attractive, the putamen may be activated due to the rewarding aspects of the product, such as its esthetic appeal, and the anticipated pleasure of the product’s use. The putamen will help to assess the subjective value or desirability of different stimuli, which can influence decision-making. Highly attractive products may activate the putamen more strongly because they are perceived as holding higher value, which in turn can drive positive consumer behaviors.

Also, the perception of esthetic pleasure can be seen as a form of reward. Aesthetic pleasure is fundamentally tied to the brain’s reward circuitry. When individuals experience beauty, it can trigger a rewarding sensation, like other pleasurable experiences such as eating or social interactions. Neuroimaging studies have shown that viewing esthetically pleasing objects can activate the putamen alongside other reward-related brain regions [17,22].

Although the putamen is not primarily responsible for processing emotions, its activity is modulated by emotional states. When experiencing esthetic pleasure, the emotional resonance of the perceived beauty can influence the activity within the putamen. This means that the emotional component of esthetic experiences is likely to enhance the putamen’s response, making the experience more rewarding.

### 5.3. Subgenual Anterior Cingulate Cortex

The subgenual anterior cingulate cortex (sgACC) is a region that is involved in emotional processing, reward anticipation, and valuation [23]. The sgACC is associated with the assessment of emotional valence, which refers to the intrinsically positive or negative quality of a stimulus that evokes affective states in an observer. The sgACC is part of the limbic system, which is crucial for emotion processing. It is thought to contribute to the generation and regulation of emotional states [24]. The sgACC is especially linked to the experience of positive emotions. When individuals encounter highly attractive products, the positive feelings elicited by the attractiveness can activate the sgACC because the brain is registering a rewarding stimulus. When individuals view highly attractive products, the sgACC may be engaged due to the positive emotions elicited by these products. Also, the sgACC is involved in determining the reward value of stimuli [25]. As mentioned before, highly attractive products may be perceived as more rewarding, thus engaging the sgACC to a greater extent than less attractive products. Similarly, the sgACC is also part of the reward system. When viewing esthetically pleasing products, positive emotional states will be induced and thereby activate sgACC.

### 5.4. Limitations

Although this study provided valuable insights into the neural correlates of product attractiveness, there are several limitations that must be acknowledged. Firstly, the sample size of our study was relatively small, which limits the statistical power of our findings and may not be representative of the population at large. This restricts our ability to generalize our results. Future studies with larger sample sizes are needed.

Moreover, we explored the neural basis of product attractiveness, but we only confined it to a single type of product. Consequently, our findings may not be applicable to other product types with different features, uses, or consumer expectations. This narrow focus may limit the breadth of our conclusions and suggests a need for caution when attempting to extrapolate our results to other product types. Also, we only focused on the group-level brain activities in response to product attractiveness but not those at the individual level. Further studies should be employed with different demographic groups and to understand the individual differences in preferences for different product designs.

We had external raters rate the attractiveness of the products instead of asking the participants to rate them inside the scanner. This may imply we could not capture real-time, subjective consumer responses, which may have provided additional insight into the cognitive and emotional processes underlying the observed neural activity. The lack of subjective data precludes a more comprehensive understanding of how participants are experiencing the product, and future studies may benefit from incorporating such measures.

In light of these limitations, the results of the current study should be interpreted with caution. We expect that the study offers a valuable preliminary exploration into the neural responses associated with these products.

## 6. Conclusions

In conclusion, our study has provided novel insights into the neural correlates of product attractiveness. The results indicated that viewing highly attractive products elicits significant activations in brain regions associated with affective value and reward processing. We found that the amygdala, the putamen, and the sgACC showed more activation when viewing highly attractive products than less attractive products. This suggests a strong emotional response to highly attractive products and that attractive products are not only more appealing but could also have a greater potential to influence consumer habits. These findings allow us to understand the neural basis of value-based decision-making and the importance of product esthetics that could enhance consumer engagement. This study adds to the burgeoning field of neuromarketing by elucidating the brain regions activated by product attractiveness. Our findings confirm that highly attractive products not only capture attention but also engage a network of brain regions implicated in emotional processing, reward anticipation, and cognitive functions.

## Figures and Tables

**Table 1:** The average scores for each stimulus among the three groups of raters.

Chair Samples		Expert Average	Community Rater	Participant Average
S1	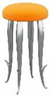	2.375	2.76	2.53
S2	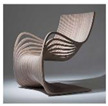	3.25	4.36	4.36
S3	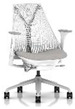	4.125	3.56	3.73
S4	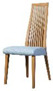	3.5	3.26	3.24
S5	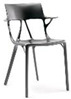	3.875	3.5	3.6
S6	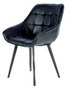	4	3.56	3.77
S7	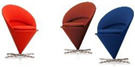	2.75	2.88	2.89
S8	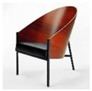	3.75	3.35	3.27
S9	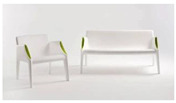	4	3.47	3.73
S10	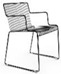	2.75	2.76	2.76
S11	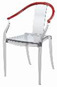	2.875	2.71	2.87
S12	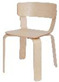	2.125	2.29	2.11
S13	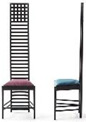	2.625	2.56	2.36
S14	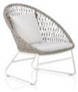	3.5	3.62	3.73
S15	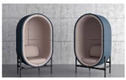	3.5	3.29	3.6
S16	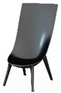	1.625	2.5	2.13
S17	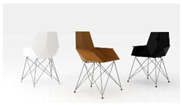	3.875	3.44	3.36
S18	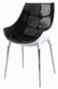	3.75	3.59	3.69
S19	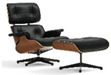	4.125	4.21	4.29
S20	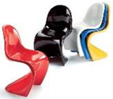	2.75	2.76	2.62
S21	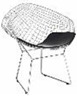	2.5	2.79	2.93
S22	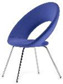	2.75	2.82	2.6
S23	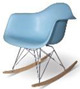	3.5	3.55	3.68
S24	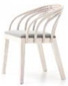	2.75	2.74	2.87
S25	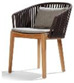	3.375	3.38	3.53
S26	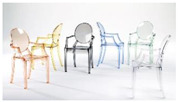	2.875	2.71	2.82
S27	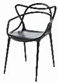	2.25	2.88	2.84
S28	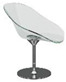	1.875	2.85	2.78
S29	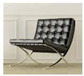	4.125	3.97	4.07
S30	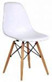	2.625	2.88	2.8

**Table 2:** (**a**). The correlation of the ratings in the three groups of raters for the highly attractive pictures. (**b**). The correlation of the ratings in the three groups of raters for less attractive pictures.

(**a**)			
	Expert	Community	Participant
Expert	1	0.815	0.856
Community	0.815	1	0.973
Participant	0.856	0.973	1
(**b**)			
	Expert	Community	Participant
Expert	1	0.815	0.856
Community		1	0.973
Participant			1

**Table 3:** Significant activation clusters.

Cluster	Voxels	Max T-Value	X (mm)	Y (mm)	Z (mm)	Label
5	222	14.7	32	−4	−22	Amygdala
4	174	10.3	−32	−10	−20	Putamen
3	121	13.3	−6	16	−18	Subgenual ACC
2	95	9.45	−26	6	48	Dorsolateral Prefrontal Cortex
1	65	10.1	44	12	−30	Temporal Pole

## Data Availability

The datasets generated and/or analyzed during the current study are not publicly available due to privacy but are available from the corresponding author upon reasonable request.
